# *V-Myc* Immortalizes Human Neural Stem Cells in the Absence of Pluripotency-Associated Traits

**DOI:** 10.1371/journal.pone.0118499

**Published:** 2015-03-12

**Authors:** María José Pino-Barrio, Elisa García-García, Pablo Menéndez, Alberto Martínez-Serrano

**Affiliations:** 1 Department of Molecular Biology and Center of Molecular Biology “Severo Ochoa” (CBMSO), Universidad Autónoma de Madrid—Consejo Superior de Investigaciones Científicas (UAM-CSIC), Campus UAM Cantoblanco, Madrid, Spain; 2 Josep Carreras Leukemia Research Institute and Cell Therapy Program, Facultat de Medicina, University of Barcelona, Barcelona, Spain; 3 Institució Catalana de Recerca i Estudis Avançats (ICREA), Barcelona, Spain; UT Southwestern Medical Center at Dallas, UNITED STATES

## Abstract

A better understanding of the molecular mechanisms governing stem cell self-renewal will foster the use of different types of stem cells in disease modeling and cell therapy strategies. Immortalization, understood as the capacity for indefinite expansion, is needed for the generation of any cell line. In the case of *v-myc* immortalized multipotent human Neural Stem Cells (hNSCs), we hypothesized that *v-myc* immortalization could induce a more de-differentiated state in *v-myc* hNSC lines. To test this, we investigated the expression of surface, biochemical and genetic markers of stemness and pluripotency in *v-myc* immortalized and control hNSCs (primary precursors, that is, neurospheres) and compared these two cell types to human Embryonic Stem Cells (hESCs) and fibroblasts. Using a Hierarchical Clustering method and a Principal Component Analysis (PCA), the *v-myc* hNSCs associated with their counterparts hNSCs (in the absence of *v-myc*) and displayed a differential expression pattern when compared to hESCs. Moreover, the expression analysis of pluripotency markers suggested no evidence supporting a reprogramming-like process despite the increment in telomerase expression. In conclusion, *v-myc* expression in hNSC lines ensures self-renewal through the activation of some genes involved in the maintenance of stem cell properties in multipotent cells but does not alter the expression of key pluripotency-associated genes.

## Introduction

Tissue specific multipotent stem cells, patient-derived induced Pluripotent Stem Cells (iPSCs) and autologous Adult Stem Cells (ASC) have enormous clinical potential for the research and treatment of degenerative diseases and genetic disorders. Proliferation of primary cells and safety concerns remain substantial roadblocks to overcome, exemplified by the heterogeneity of the cell populations, the potential for genetic and epigenetic abnormalities, the tumorigenicity, and the immunogenicity of transplanted cells [1]. Thus, understanding the mechanisms controlling stem cell self-renewal is critical for boosting the efficiency and safety of stem cells in clinical research.

Complete reprogramming to pluripotency comprises intermediate stages between the initiation of reprogramming and the stabilization of pluripotency. The cells in these intermediate stages could be useful for many therapeutic indications, particularly in terms of safety [2,3]. In this context of intermediate precursors, it was long ago hypothesized that *v-myc* immortalized human Neural Stem Cells (hNSCs) could represent more de-differentiated cells [4,5].

*V-myc* is the viral homolog of c-Myc, initially identified in an acute avian retrovirus (MC29), that belongs to a family of transcription factors that are able to bind to approximately 10–15% of the genome [6], controlling many cellular processes: it stimulates cell proliferation and stemness, represses differentiation and *ergo*, connects cell cycle regulation to the maintenance of pluripotency.

In addition, it is well established that *v-myc* promotes an unlimited proliferation and stability of neural progenitor’s properties in the absence of long-term transformation [7,8]. Furthermore, the regulatable expression of *v-myc* maintains a safe, effective and efficient self-renewing state [9]. Taking these results into account we hypothesized if the stable expression of *v-myc* could theoretically originate an intermediate population of more de-differentiated progenitors.

Deciphering the role of MYC is therefore important for understanding the maintenance of stemness and pluripotency. To date, c-Myc has been shown to boost the initial reprogramming by upregulating its targets during the first wave of the reprogramming process [10]. However, it fails to completely reprogram to pluripotency on its own [11–16]. Several hypothesis propose different roles of the Myc family of transcription factors in partial reprogramming and in the enhancement of cell renewal [17]: 1) Myc could be implicated in the selection of a rare population of cells with predetermined traits to undergo pluripotency [18]; 2) Myc could probably modify epigenetic patterns inducing changes in chromatin [19,20] 3) Myc could promote the de-differentiation or blockade of additional differentiation [21]; 4) Myc could induce a cell cycle program specifically needed for self-renewal, by accelerating cell cycle progression (activating cyclins and inhibiting cyclin-dependent kinases (Cdk) activity [22] and increasing telomerase activity [8,15]). Moreover, Myc’s ability to immortalize cell cultures probably helps the progression of reprogramming, as it has been shown that immortalization and indefinite propagation is one of the essential features of reprogrammed-like cells [23].

In summary, as it has been previously proposed, *v-myc* mediated immortalization could not only promote cell division, but could also return hNSCs to an earlier developmental state. One therefore may hypothesize that *v-myc* immortalized hNSCs could acquire traits of pluripotency. In this study, we investigated whether a single transgene, *v-myc*, ectopically expressed in hNSCs may, in addition to sustaining cellular self-renewal, induce an intermediate, more de-differentiated state.

## Materials and Methods

### 1. Cell Culture

#### 1.1 Bioethics

All the cells originated from tissues donated for research after written informed consent, and following European Union (EU) directives, the declaration of Helsinki, ethical guidelines of the European Network for Transplantation (NECTAR) and Spanish Biomedical Research Law, July 2007. All procedures were also approved by the Bioethics of Research Committee of the University Autónoma de Madrid (Spain), the Ethics Committee for Clinical Research of the local Government of Madrid (CEIC-Regional), and the national authority (Instituto de Salud Carlos III (Spain)).

#### 1.2 Cell lines of hNSCs

We have used the hNS1, hCTX, hVM1 cell lines. Cell isolation and immortalization have been previously described [7, 8, 37–40]. For experiments, cells were seeded at 10,000 cell/cm^2^(unless otherwise indicated) on poly-L-lysine (10μg/ml; Sigma-Aldrich) coated plastic or poly-L-lysine (25–50 μg/ml) glass coverslips with HSC medium, a chemically defined medium [Dulbecco’s Modified Eagle Medium (DMEM):F12 (1:1) with GlutaMAX-I medium (Gibco) containing 1% AlbuMAX (Gibco), 50 mM Hepes (Gibco), 0.6% D-Glucose, 1% N2 supplement (Gibco), 1% non essential amino acids mixture (NNEA), and 1% penicillin-streptomycin (P/E)]. The HSC medium was supplemented with Epidermal Growth Factor (EGF) and basic Fibroblast Growth Factor (bFGF) (20 ng/ml, R&D system) to promote cell proliferation. Cells were maintained at standard normoxic culture conditions proliferated at 37°C, and 95% humidity in a 5% CO_2_ and normal atmospheric oxygen levels (20% O_2_) except for hVM1 cells that were incubated in a 5% O_2_–5% CO_2_ levels (dual CO_2_/N_2_ incubator, Forma). Samples for analyses were collected at least 48h following the last passage by trypsinization.

#### 1.3 Non-immortalized forebrain human Neural Progenitor Cells (hNPCs)

Neurospheres were obtained from forebrain tissue derived from aborted human fetuses of 9.5 and 10 weeks gestational age (Lund University Hospital/ Wallenberg Neuroscience Center; Dr. Bengt Juliusson; Lund, Sweden). Neurospheres were grown in suspension in HSC medium supplemented with EGF and bFGF (20 ng/ml, R&D system) and heparin (5 μg/ml) and passed twice by mechanical protocols. Cells were maintained in standard normoxic culture conditions. When used for experimentation, they were seeded as adherent cultures (10,000 cells/cm^2^) on poly-L-lysine (10μg/ml; Sigma-Aldrich) coated plastic or poly-L-lysine (25–50 μg/ml) glass coverslips also treated with laminin (2,5 μg/ml) in proliferation conditions.

#### 1.4 Human foreskin Fibroblast cell lines

The commercial hFF-1 line was used (American Type Culture Collection, LGC Standards, SCRC-1041TM). Cells were proliferated in standard normoxic culture conditions. Cells were seeded at 10,000 cells/cm^2^ on coated plastic previously treated with 0,1% Gelatin (Merck) in Phosphate Buffered Saline (PBS) or glass coverslips treated with 0.2% Gelatin in PBS. The culture medium consisted in DMEM (Gibco), 10% FBS and 1% L-Glutamine. When used as feeders, mitomycin C was added (7μg/ml or 1μg/ml, 3 hours or overnight, respectively) when the cells were confluent. After that, medium was changed every three days until used as feeders.

#### 1.5 Embryonic stem cell lines

In the present study we have used representative hESC lines such as HS181 [41] and H9 [42]. The cells were seeded in a confluent conditioned fibroblast medium, and maintained in standard normoxic culture conditions. The cells grew as colonies in a hESC medium [80% Knockout DMEM (Gibco), 20% Serum Replacement (Gibco), 200 mM L-Glutamine, 1% NNEA, 1% P/E, 0,1 mM of β-Mercapto Ethanol, and bFGF (final concentration of 4ng/ml, R&D system)]. Human ESCs were split upon treatment with Accutase (PAA LabClinics), scratched and gently aspirated with a p10 pipette avoiding removing the hFF-1 feeder layer. Next, they were centrifuged during 5 min at 55g and seeded at 10,000 cells/cm^2^ onto a fresh hFF-1 feeder layer.

### 2. RNA Isolation and Real Time-Quantitative-Polymerase Chain Reaction (RT-Q-PCR)

Total RNA was extracted using the High Pure RNA isolation Kit (Roche) following manufacturer’s guidelines. Total RNA was then quantified by absorption at 260 nm in a Nanodrop spectrophotometer. Samples with 260/280 and 260/230 ratios in the range of 1.8–2 and 2–2.35, respectively, were accepted. Microfluidic RT-Q-PCR was performed in triplicate using the TaqMan Human Stem Cell Pluripotency Array (Applied Biosystems). Regular RT-Q-PCR was performed as previously described [43] using the ABI PRISM 7900 HT Sequence Detection System. One μg of RNA for each sample was subjected to reverse transcription using the High Capacity cDNA Achieve Kit (Applied Biosystems). For Real Time-Q-PCR 0.5 μg cDNA was amplified using TaqMan based technology (Applied Biosystems). The expression data were normalized taking into account the values of the genes that were expressed in all samples. The normalization formula is as follows: ΔCt(test) = (Ct(gene)-Ct(total average))/SD(Ct(total)).

### 3. Immunocytochemistry (ICC)

Two days following the last passage, the cultures were rinsed with 0.1 M PBS and fixed for 15 min in freshly prepared 4% Paraformaldehyde/Phenylalanine (PFA/PHE) /4% sucrose. Cultures were blocked for 1h at room temperature in 10% normal horse serum or goat serum, 0.25% Triton X-100 in PBS (absent for surface proteins) and incubated overnight at 4°C with primary antibodies (dissolved in 1% serum and 0.25% Triton X-100 in PBS) against SSEA-1 (1:100, mouse mAb, Hybridoma Bank MC480), SSEA-4 (1:100, mouse mAb, Millipore MAB 34304), Tra 1–60 (1:100, mouse mAb, Millipore MAB 4360), and Tra 1–81 (1:100, mouse mAb, Millipore MAB4381, Nanog (1:100, goat pAb, R&D Systems AF1997), Oct3/4 (1:100, rabbit pAb, Santa Cruz Biotechnology SC9081), Sox2 (1:100, rabbit mAb, Millipore AB5603), and Alkaline Phosphatase (AP) (1:50, mouse mAb, R&D Systems MAB1448). After removal of the primary antibodies, cultures were incubated with the secondary antibodies as FICT-conjugated antibody (Alexa 488, 1:500, goat anti rabbit IgG, Molecular Probes A11034), Cy3-conjugated antibodies (Texas Red, 1:500, goat anti mouse IgM, Vector Laboratories TI-2020; Alexa 594, 1:500, chicken anti goat IgG, Molecular Probes a 21468; Cy3, 1:500, goat anti mouse IgG, Jackson Inmuno Research Lab., Inc. 111-165-003). Appropriate controls included negative controls where primary antibodies were omitted, and positive controls were hESCs, known to express the antigens under study. Nuclei were counterstained with Hoechst 33258 (Molecular Probes) and mounted with Mowiol (Polysciences 17958) and analyzed. Microscopic examination and photography of specimens were performed.

### 4. Alkaline Phosphatase Activity Analysis

The AP activity assay was performed according to the manufacturer’s instructions (Cell Biolabs, Inc., San Diego, CA). The activity was determined by measuring A_405_ and normalized against the amount of protein in the assay. For quantification, three biological replicates were used for each sample. The enzyme activity was expressed in international units (μmol of product produced per minute and per μgram of protein; μmol x min^−1^ x μg^−1^).

### 5. Image Analysis

Analysis and photography of fluorescent cultures were done using a vertical fluorescence microscope Axioskop2 plus (Zeiss) coupled to a Coolsnap FX color camera navigated by Metavue 5.07 (Universal Imaging) operated by Adobe Photoshop 5.0 (Pantone Inc). Images have been processed using the Adobe Design Premium CS5.

### 6. Statistical Analysis

General statistical analyses were run using Statistica *vs7* (StatSoft.Inc. Tulsa OK). Results are shown as the average ± Standard Error of the Mean (S.E.M.) of data, from four samples and from four experiments, unless stated otherwise.

### 7. Bioinformatics Tools

The data discussed in this publication has been deposited in NCBI’s Gene Expression Omnibus (GEO) and are accessible through GEO Series accession number GSE63710.

#### Clustering Analysis

Clustering of data was done with the program Gene Cluster vs. 3.0 using a Hierarchical clustering single linkage method. The distance measures were taken based both on the Pearson correlation (centered) and Euclidean distance and both methods gave the same results. Images of Tree Clustering were done by JavaTreeView vs. 1.1.6.

#### Principal Component Analysis

This analysis was done with 32 principal components, with the R Program (R Project for Statistical Computing vs. 2.13.0). The first three components were taken into account as are the ones that most contribute to the differences. Images of the tridimensional graphic were prepared with Adobe PostScript CS5.

## Results

To address the question of whether *v-myc* modifies the stemness and potency of hNSCs, we first studied the expression of 90 genes related to stemness, self-renewal and differentiation in several *v-myc* immortalized cell lines (hNS1, hVM1, hCTX), non-immortalized neurospheres (hNPCs derived from 9.5 and 10 weeks-old fetuses), hESCs (H9 and HS181) [24,25] and human foreskin fibroblasts (hFF-1).

**Fig 1:**
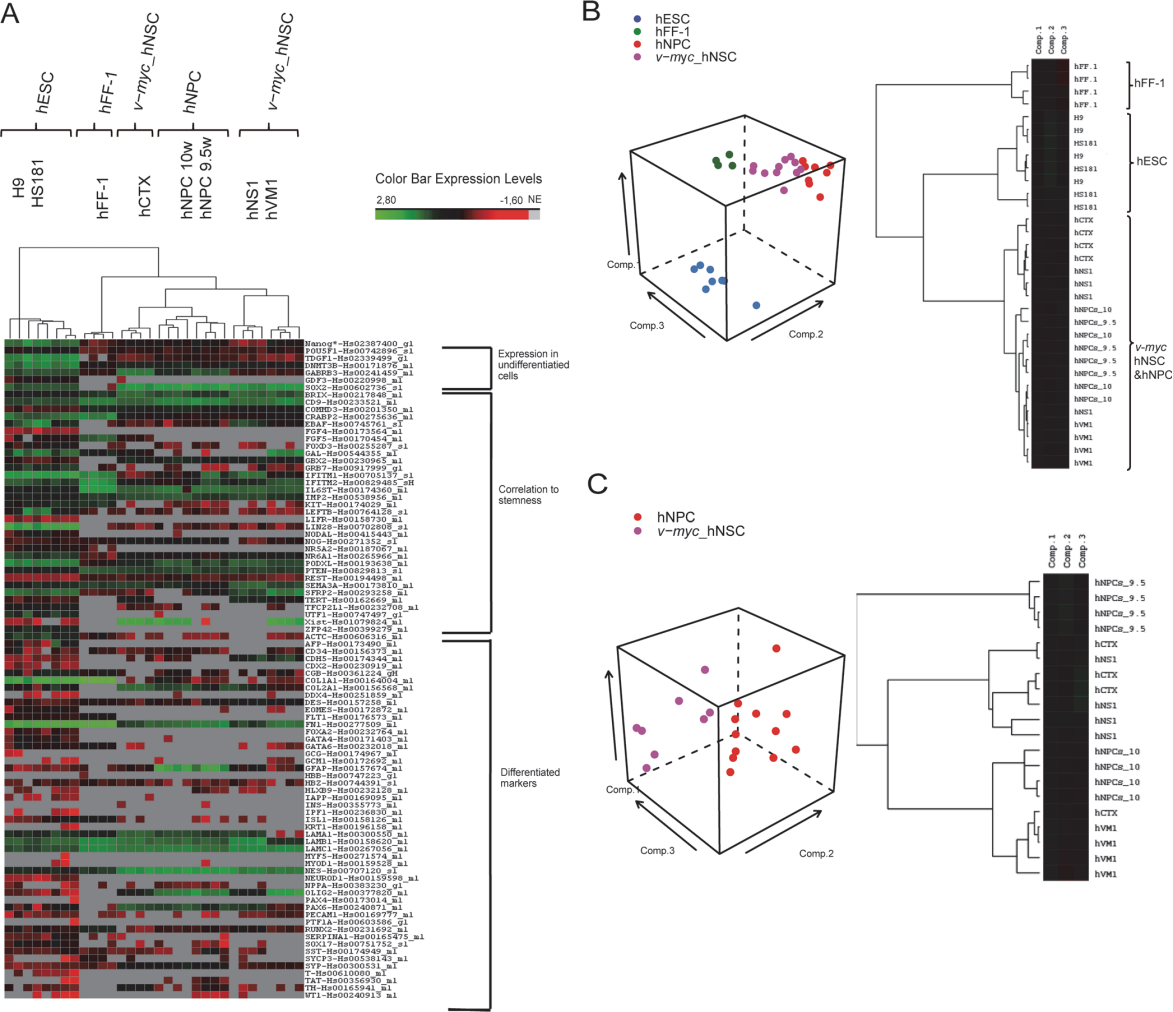
Gene expression pattern of the different cell lines, clustering and PCA analysis. A) The array data were normalized and a hierarchical clustering was run. On top of the heatmap, the dendrogram shows the clustering among the different samples. The classified list of the genes under study appears on the right side of the heatmap. The Color Bar Expression Levels show the differences in gene expression: black means no differences, green means a high expression, and red means low expression. Grey color mean No Expression (NE). B) Human Stem Cell Pluripotency Array (TaqMan) gene expression data from the different studied cell lines were subjected to a PCA. The graph presents the first, second and third components. For every cell line, the dots represent the value of each sample. To confirm the results of the PCA, a clustering analysis with the dataset of the components was done (right side of the panel). C) PCA analysis of *v-myc* immortalized cells and non-immortalized hNPCs and the clustering of the PCA data to confirm the similarity between *v-myc* immortalized and the hNPCs neurospheres according to the three components.

A hierarchical clustering was performed to group the cell lines under study in accordance with their gene expression profiles (Fig. 1A). The analysis identified two main clusters which segregate one from each other (that is, with the longest significant distance). Human ESCs (hESCs) clustered together and segregated from the remaining cell lines, which comprised another cluster. Within this second cluster, hFF-1 grouped independently from neural cells. Regarding neural cells, hNPCs and *v-myc* immortalized cortex cell line (hCTX) clustered separately from *v-myc* immortalized ventral midbrain cell line (hVM1) and *v-myc* telencephalon/diencephalon cell line (hNS1) (Fig. 1A). Thus, the hierarchical cluster analysis groups the *v-myc* immortalized cells closer to hFF-1 than to hESCs. Attempts to separately cluster the non-immortalized hNPCs and the *v-myc* immortalized hNSC cell lines using this analysis were negative, indicating a high degree of similarity.

Then, the gene expression data were analyzed by Principal Component Analysis (PCA) to verify gene expression differences and generate discrete groups among the studied cell lines, according to the genes that contributed most to each separation (component). The three principal components explained 60% of the dataset variability. The first component contributes with 43% of the variability, and sorted the hESCs (H9 and HS181) out of the rest of the groups. The second component contributes with 11% of the variability, which separated the fibroblasts (hFF-1). Finally, the third component minimally separated the *v-myc* immortalized hNSCs from their non-immortalized counterparts (neurospheres, hNPCs) with a variability of only 6% (Fig. 1B). Among the genes that substantially contributed to the differences between *v-myc* immortalized hNSCs and neurospheres is the hTERT (Telomerase Reverse Transcriptase).

We next proceeded to perform a Principal Component Analysis of immortalized cells *vs*. hNPCs. The three principal components contributed with 71% of the variability of the dataset. The contribution of each component was 56%, 9% and 6% respectively (Fig. 1C). In order to confirm both PCA results, a clustering analysis with the dataset of the components was performed for the PCA of the four cell lines (hESCs, hFF-1, hNPCs, and *v-myc* immortalized cells) (Fig. 1B) and for the PCA of the *v-myc* immortalized cells and hNPCs (Fig. 1C). The clustering of the PCA-dataset of the four cell lines confirmed the clustering previously done with the normalized data, in which three groups cluster separately: hESCs, hNSCs and hFF-1 (Fig. 1B). However, the clustering of the PCA-dataset of the *v-myc* immortalized cells and hNPCs did not separate these two cell types (Fig. 1C). Therefore, the PCA of the *v-myc* immortalized cells and hNPCs (non-immortalized neurospheres) demonstrate that there are not significant gene expression markers capable of segregating the neural precursor/stem cell group based on pluripotency and stemness.

In summary: firstly, the hierarchical clustering analysis unambiguously placed immortalized *v-myc* cell lines and non-immortalized cell lines within the same group. Secondly, both cell types were more related to a differentiated state, exemplified by hFF-1, than to an undifferentiated state, represented by hESCs. Thirdly, the PCA analysis, comparing the four cell lines, only found a variability between the *v-myc* immortalized cell lines and the non-immortalized counterparts of 6%. According to these gene expression results, we suggest that the state of immortalized *v-myc* cell lines is undistinguishable from that of non-immortalized cell lines.

**Fig 2:**
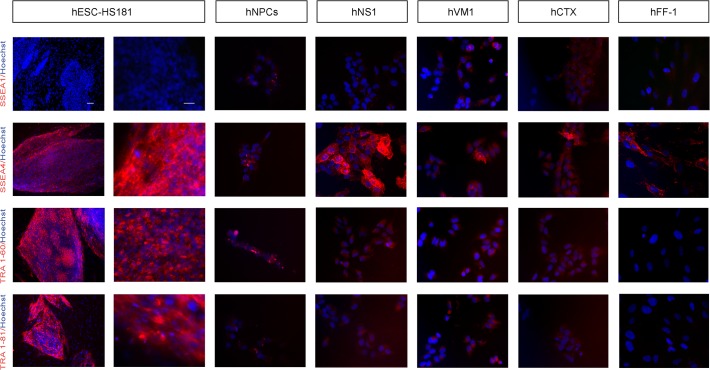
Immunocytochemistry of stemness surface markers. Staining was performed 48 hours following the last passage. From left to right: staining displayed by hESCs (HS181 line, low and high magnification), hNPCs, *v-myc* immortalized lines (hNS1, hVM1, and hCTX) and fibroblasts (hFF-1). From top to bottom the markers studied were: SSEA1, SSEA4, TRA1–60 and TRA1–81. Hoechst 33258 nuclear staining is shown in blue. Scale bar represents 100 μm for hESCs in the left hand column (low magnification) and 25 μm for all the other microphotographs.

**Fig 3:**
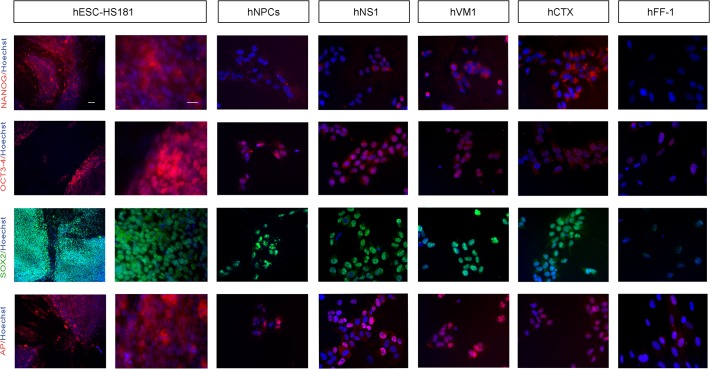
Immunocytochemistry of pluripotency markers. Staining was performed 48 hours following the last passage. From left to right: staining displayed by hESCs (HS181 line, low and high magnifications), hNPCs, *v-myc* immortalized lines (hNS1, hVM1, and hCTX) and fibroblasts (hFF-1). From top to bottom the markers studied were: NANOG, OCT3/4, SOX2 and AP. Hoechst 33258 nuclear staining is shown in blue. Scale bar represents 100 μm for hESCs in the left hand column (low magnification) and 25 μm for all the other microphotographs.

A second approach to assess our question of whether *v-myc* returns hNSCs to an early state of de-differentiation was to study the standard pluripotency-related cell markers [26] by immunocytochemistry in the *v-myc* immortalized lines in comparison to hESCs and hFF-1. The markers studied included the surface hESC markers identified as the globoseries glyocolipid antigens SSEA4 and SSEA1 (Stage-Specific Embryonic Antigens) and the keratin sulphate antigens TRA1–61 and TRA 1–81. Considering the staining intensities of hESCs (positive control) and hFF-1 (negative control), the *v-myc* immortalized cells displayed only traces/residual staining for TRA 1–60 and TRA 1–81 (Fig. 2). hNS1 cells showed some expression of SSEA4, whereas only occasional cells were positive in hVM1 and hCTX. For the case of SSEA1, a marker positive in committed cells, some signal is observed in hVM1 and hCTX, which are tissue specific NSCs. Notably, the level of staining for these markers in the non-immortalized hNPCs does not differ from that observed in the immortalized lines (Fig. 2). A second set of tested markers included NANOG (Nanog homeobox), POU5F1 Octomer-4 (POU class 5 homeobox 1, formely known as OCT3/4,) SOX2 (member of the family of Sex determining region Y Box 2), and AP (Alkaline Phosphatase). In relation to NANOG, OCT3/4, SOX2 and AP, varying degrees of staining were observed in the immortalized cells, making it difficult to draw any firm conclusion. Whereas *v-myc* cells weakly stained for NANOG ICC, the cells did show a positive stain for the other markers (Fig. 3). Similarly to the surface markers in Fig. 2, the level of staining for NANOG, OCT3/4, SOX2 and AP in the non-immortalized hNPCs does not substantially vary from that observed in the immortalized lines.

**Fig 4:**
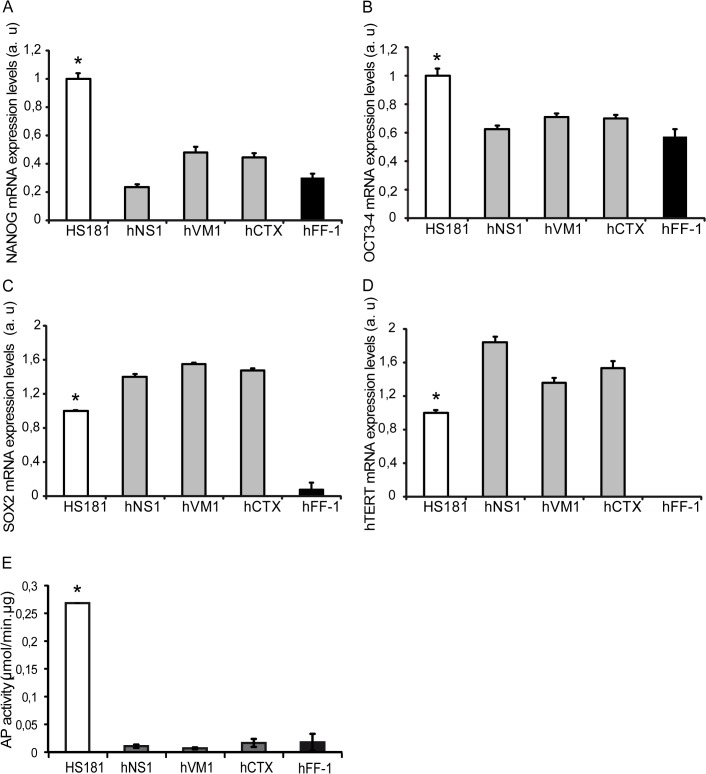
Expression of pluripotency markers and quantification of alkaline phosphatase activity. mRNA levels were quantified by RT-Q-PCR. NANOG (A), OCT3–4 (B), SOX2 (C) and hTERT (D) expression in the *v-myc* immortalized cell lines shows significant differences with the HS181 pluripotent cell line. Data are the average ± S.E.M (n = 4). * P<0.05; one-way ANOVA, followed by a *post-hoc* LSD and Bonferroni test or non parametric Kruskal-Wallis and median test followed by a *post-hoc multiple comparisons of mean ranks for all groups*. E) Quantification of alkaline phosphatase activity in the cell lines. Data are the average ± S.E.M (n = 3). * P<0.05; non parametric Kruskal-Wallis and median test followed by a *post-hoc multiple comparisons of mean ranks for all groups*.

In order to clarify the level of expression of NANOG, OCT3/4, SOX2, and that of the catalytic subunit of telomerase, hTERT, their mRNA expression was quantified in *v-myc* immortalized cells in comparison to hESCs. As shown in Fig. 4A and B, considering 100% of expression in HS181 cells, *v-myc* immortalized cells express on average 39% of NANOG, and 68% of OCT3/4. On the other hand, *v-myc* immortalized cells express on average 147% of SOX2 and 158% of hTERT (Fig. 4C and D).

Regarding AP, we performed an enzyme activity assay to correlate the positive histochemical staining pattern with the actual enzymatic activity. The measurement of this activity in quantitative terms, (Fig. 4E) indicated that HS181 cells are endowed with a high AP specific activity (0.26±0.52x10^−3^ μmol/min. μg, n = 3) compared with the very low activity of *v-m*yc immortalized cells (hNS1 = 0,01±2,82x10^−3^ μmol/min. μg, n = 3; hVM1 = 6,73x10^−3^±1.63x10^−3^ μmol/min. μg, n = 3; hCTX = 16.5x10^−3^±7.12x10^−3^ μmol/min. μg, n = 3) and fibroblasts (17.7x10^−3^ ±15.1x10^−3^ μmol/min. μg, n = 3). Both experiments clarify that *v-myc* immortalized cells express less NANOG and OCT3/4, and more SOX2 and hTERT when compared to hESCs, and that AP activity is negligible in *v-myc* immortalized cells in comparison to hESCs. In sum, these results confirm that *v-myc* does not induce the expression of pluripotency markers or traits in comparison to those of hESCs.

## Discussion

In the field of hNSCs research, the process of self-renewal is understudied, the question of the molecular mechanisms by which *v-myc* immortalizes the cells still remains unanswered, and the exact meaning of immortalization, apart from indefinite expansion, is poorly understood. Our results help to clarify if *v-myc* exerts any effect on de-differentiating hNSCs from a multipotent state back to a more pluripotent state, similar to that of hESCs. Transcription factor-induced reprogramming to pluripotency is a gradual and multistep process, with different stages of reprogramming towards pluripotency. Early key events associated with reprogramming are AP expression, followed by SSEA-1 silencing, and, later on, the activation of the endogenous OCT3–4 and NANOG genes, key pluripotency regulators [2,3]. MYC has been shown to be crucial for the loss of the somatic expression program, whereas OCT3–4, KLF-4 and SOX2 (OKS) induce the expression of pluripotency-associated genes [27]. The expression of telomerase (TERT) and the silencing of the X chromosome in female cells also correlate with the time window when cells enter a self-sustaining pluripotent state. Aside from the differences in the expression of pluripotency-related genes and activation of lineage-specific genes that are not expressed in fully pluripotent stem cells [28], these intermediate cell populations usually show differences in morphology. Neural stem cells endogenously express SOX2, c-MYC, KLF4, and iPSCs have been derived from NSCs with OCT3–4 alone [29]. OCT3–4 seems to be the essential reprogramming factor for most initial cell types [30] but SOX2 synergistically activates the Oct-Sox axis and enhances OCT3/4, regulating the expression of pluripotent stem-cell-specific genes [31].

Based on these studies, we hypothesized that *v-myc* immortalization could turn the cells into a more undifferentiated state. However, our expression analysis demonstrates that the *v-myc*-mediated immortalization rendered hNSCs to a very similar (non-pluripotent state) to that shown by their multipotent counterparts in the absence of an exogenously expressed *Myc*.

Previously, we already described that hTERT, is quickly and directly upregulated by *v-myc* in immortalized cells [8], and these results have been confirmed in the present study. Interestingly, among several pluripotency-related genes, hTERT is the only one modulated by *v-myc*, consistent with the results of the cluster analysis performed (Fig. 1). This result indicates that *v-myc* hNSCs do not substantially differ from their non-immortalized counterparts, and at the same time indicates that they are not similar to pluripotent cells. Furthermore, it seems that hTERT activation may be a mechanism through which *v-myc* (and probably c-Myc) immortalization acts [32]. This result is consistent with a recent study showing that high levels of *Myc* expression are important for sustaining the self-renewal properties of partial iPSCs and of ESCs [33].

Lastly, the fact that *v-myc* immortalized hNSCs do not show traits of pluripotency actually constitutes an advantage. It has been extensively demonstrated that *v-myc* immortalized cells are not transformed, and therefore, concerns and debates about their safety remain speculative [34–36]. Indeed, not sharing a molecular profile with pluripotent cells eliminates one further concern, that of the possibility for tumor formation shown by hESCs and hiPSCs.

In conclusion, *v-myc* activates an immortalization program leading to the maintenance of cell self-renewal and inhibition of cellular senescence yielding perpetual hNSC lines. This can be easily explained by the activation of telomerase gene expression ([8] and present work). However, *v-myc* induces these effects without inducing pluripotency-associated traits. It only activates the expression of one gene involved in sustaining stem cell properties in multipotent cells (such as hTERT). Further studies are needed to clarify the molecular basis of immortalization of hNSCs by *v-myc*, in order to understand how to indefinitely propagate hNSCs in its absence, replacing it with alternative factor(s).
